# Genomic selection for tar content in *Nicotiana tabacum*: genetic architecture analysis and model evaluation

**DOI:** 10.3389/fpls.2026.1721129

**Published:** 2026-03-17

**Authors:** Linjie Guo, Bo Kong, Hui Chen, Min Ren, Lirui Cheng, Aiguo Yang, Lei Liang, Yanjun Zan, Huan Si, Changchun Cai

**Affiliations:** 1 College of Agronomy, Qingdao Agricultural University, Qingdao, China; 2 Institute of Tobacco Research, Chinese Academy of Agricultural Sciences, Qingdao, China; 3 Technical Center, Hunan Tobacco Industry Co., Ltd., Changsha, China; 4 Hubei Provincial Tobacco Quality Supervision and Inspection Station, Wuhan, China; 5 State Key Laboratory of Maize Bio‐breeding, Frontiers Science Center for Molecular Design Breeding, National Maize Improvement Center, College of Agronomy and Biotechnology, China Agricultural University, Beijing, China; 6 Joint International Research Laboratory of Crop Molecular Breeding, Frontiers Science Center for Molecular Design Breeding, National Maize Improvement Center, College of Agronomy and Biotechnology, China Agricultural University, Beijing, China; 7 Department of Plant Physiology, Umeå Plant Science Center and Integrated Science Lab, Umeå University, Umeå, Sweden; 8 Tobacco Research Institute of Hubei Province, Wuhan, China

**Keywords:** genome-wide association analysis, genomic selection, model evaluation, tar content, tobacco

## Abstract

**Introduction:**

Despite its economic importance, reducing tobacco tar content remains challenging due to its complex genetic basis.

**Methods:**

Here, we evaluated 436 diverse tobacco accessions to characterize the genetic architecture of tar content and develop an optimized genomic selection strategy. Based on these findings, sixteen genomic prediction models were assessed using five-fold cross-validation.

**Results:**

Genome-wide association analysis detected no major-effect loci, and regional heritability mapping revealed localized enrichment of small-effect variants, particularly on chromosome 17, indicating a predominantly polygenic architecture. rrBLUP achieved the highest prediction accuracy (0.84) with superior computational efficiency, followed closely by GBM (0.83). The robustness of rrBLUP was further confirmed in an independent panel of 36 accessions (Pearson *r* = 0.888).

**Discussion:**

Together, our results demonstrate that tobacco tar content is governed by dispersed small-effect loci with regional aggregation and establish rrBLUP as a robust and practical model for genome-wide prediction, providing methodological guidance for low-tar tobacco breeding.

## Introduction

Despite the high economic value of tobacco, growing public awareness of its associated health risks has heightened concerns over tar content in cigarettes (Frost et al., 1995; Zhao et al., 2020). Reducing tar and its related harmful constituents has therefore become a primary objective in the tobacco industry. Tobacco tar is a complex mixture containing various carcinogens and toxic compounds, which can be broadly grouped into organic carcinogens (e.g., polycyclic aromatic hydrocarbons such as benzo[a]pyrene, tobacco-specific nitrosamines, and phenolic compounds), heavy metals (e.g., cadmium and arsenic), and other harmful constituents. Although these compounds occur only in trace amounts, their cumulative effects pose significant health risks. Consequently, minimizing tobacco tar content to mitigate potential harm has emerged as a global consensus and aligns with the industry’s commitment to public health.

Linkage and association mapping approaches, using either artificial or natural populations, have achieved considerable success in elucidating the genetic basis of key traits in tobacco and other crops (Yano et al., 2016; Sun et al., 2017; Ma et al., 2021; Tang et al., 2021; Si et al., 2025). However, tobacco tar content, as a complex quantitative trait, presents two major challenges for genetic dissection and breeding applications. First, accurate phenotypic evaluation is labor-intensive, costly, and difficult to scale to large populations. For conventional quantitative traits such as plant height or leaf width, genetic loci can typically be mapped rapidly in early-generation populations using linkage or association analyses (Karamat et al., 2021; Zhang et al., 2021). In contrast, tar measurement requires strict control of sample size and uniformity, making it difficult to obtain sufficient material from individual plants in early generations (e.g., F_2_ populations) for reliable phenotyping. When population size increases, the cost of tar quantification rises sharply, further constraining large-scale evaluation. Second, as a chemically complex mixture, tar exhibits an intricate genetic architecture. The limited detection power of conventional mapping methods hampers the identification and validation of loci with small effects, restricting their utility in breeding programs.

Genomic selection (GS), also known as genomic prediction (Desta and Ortiz, 2014), extends the concept of marker-assisted selection to the genome-wide scale (Meuwissen et al., 2001; Goddard, 2009). GS integrates genome-wide molecular marker data with phenotypic information from a training population to build predictive models that estimate genomic estimated breeding values (GEBVs). Once the model is trained, GEBVs of offspring can be predicted using only genotypic data, enabling selection without direct phenotypic evaluation. This approach substantially reduces the labor and cost of phenotyping and is particularly advantageous for traits that are difficult or expensive to measure, such as tobacco tar content. Implementation of GS requires two distinct populations: a training population (TP) and a breeding population (BP) (Schaeffer, 2006; Daetwyler et al., 2008). Individuals in the TP are both genotyped and phenotyped to calibrate a regression model that captures genome-wide marker effects, whereas individuals in the BP are genotyped only, and their GEBVs are predicted using the trained model (Xu et al., 2020). Selection is then performed by ranking BP individuals based on their predicted breeding values, with the highest-ranking individuals retained for breeding. Initially developed in animal breeding, GS has been successfully applied in major crops such as maize (Cui et al., 2016) and rice (Huang et al., 2010), where it has substantially increased selection efficiency.

Recent studies have begun to explore GS in tobacco breeding. For example, Carvalho et al (Carvalho et al., 2022). evaluated different statistical models for predicting superior tobacco genotypes in the absence of phenotypic data, using 72 hybrids derived from 13 flue-cured tobacco inbred lines. Their results indicated that genotype-by-environment interactions significantly affected the predictive accuracy of GS. Similarly, Tong et al. (Tong et al., 2021) applied GS to predict multiple agronomic traits in a tobacco recombinant inbred line (RIL) population and systematically examined the effects of marker density, population size, and training-to-testing ratio on prediction accuracy, identifying the Bayes B model as the most accurate. Although the application of GS in tobacco remains at an early stage, these studies have established a methodological foundation for its broader utilization in tobacco improvement.

In the present study, we evaluated tar content in 436 genetically diverse tobacco accessions. Using 16 GS models combined with five-fold cross-validation, we assessed prediction accuracy and developed an optimized GS model for tar content. This model provides both a theoretical and methodological framework for reducing tar content through genome-wide selection, offering a powerful tool to accelerate the breeding of low-tar tobacco varieties.

## Materials and methods

### Experimental materials and field design

A total of 436 tobacco accessions representing broad genetic diversity were selected for this study. These materials included core varieties released or registered over the past three decades, germplasm with high breeding potential, as well as several key commercial cultivars across the world.

The experiment was arranged in a randomized complete block design with two replicates. Each plot covered an area of 22.0 m² and consisted of two rows planted at a spacing of 1.1 m between rows and 0.6 m between plants, with a row length of 10.0 m. Single plants were transplanted per hill, giving more than 20 individuals per plot. Seeds were sown in February, and mature plants were harvested in three successive stages during mid-September.

### Phenotypic identification

Following harvest, tobacco leaves were cured in accordance with the Chinese National Standard GB/T 23219-2008. To minimize the influence of leaf position and developmental stage on phenotypic variation, the middle leaves, approximately the 8^th^-12th nodes from the bottom, from each of the 436 tobacco accessions were uniformly selected based on consistent nodal position and physiological maturity. The middle leaves of 436 tobacco accessions were subsequently processed following the protocols of YC/T 549.5–2016 and GB/T 18771.2–2015 to ensure standardized cutting, sample preparation, and phenotypic consistency.

Prior to analysis, all cigarette samples were conditioned in a constant temperature and humidity chamber (22 ± 1°C, 60% ± 2% RH) for a minimum of 48 hours to equilibrate moisture content. Tar content was quantified at the Quality and Safety Research Center of the Tobacco Research Institute, Chinese Academy of Agricultural Sciences, using a standardized smoking machine. Tar content (nicotine-free dry particulate matter, NFDPM) was determined according to Chinese National Standards GB/T19609-2024, GB/T 23355 and GB/T 23203.1. After smoking, particulate matter deposited on glass fiber filters was extracted with isopropanol and quantified gravimetrically. Specifically, smoke particulate matter collected on Cambridge glass fiber filters was transferred into 44-mm conical flasks containing 20 mL of isopropanol (with internal standards for tar and moisture determination). The filters were fully immersed and shaken at room temperature for at least 20 min to ensure complete extraction. Aliquots of the extract were used for moisture determination following GB/T 23203.1, while tar content was quantified according to GB/T 23355.

Tar mass per cigarette was calculated as:


$$m_{NFDPM}=m_{TPM}-m_{W}-m_{N}$$

where *m_NFDPM_* represents tar mass (mg), *m_TPM_* is total particulate matter (mg), *m_W_* is moisture mass (mg), and *m_N_* is nicotine mass (mg). Each sample was measured in triplicate, and the mean value was used as the phenotypic measurement for downstream genetic analyses and genomic selection.

### Heritability estimation

Narrow-sense heritability (*h*²) for tobacco tar content was estimated through variance component analysis based on the genomic best linear unbiased prediction (GBLUP) framework, as expressed in the following formula:


$$h^{2}=\frac{\sigma_{ɡ}^{2}}{\sigma_{ɡ}^{2}+\sigma_{e}^{2}}$$

Here, *h²* represents the narrow-sense heritability, $\sigma_{ɡ}^{2}$ denotes the genetic variance, and $\sigma_{e}^{2}$ represents the residual variance. These variance components, together with the standard error of heritability, were estimated using the restricted maximum likelihood (REML).

### Genotype data processing

Genotyping of the 436 tobacco accessions was conducted using genotyping-by-sequencing (GBS) technology (Elshire et al., 2011; Lebedev et al., 2020). Genomic DNA was extracted using the CTAB protocol and digested with the restriction enzymes NlaIII and MseI. The resulting fragments were purified with AMPure XP beads, and sequencing libraries were constructed for high-throughput sequencing on the Illumina NovaSeq platform. Raw sequence reads were processed with fastp (Chen et al., 2018) to remove low-quality bases and adapter contamination, and the clean reads were aligned to the *Nicotiana tabacum* L. var. ZY300 reference genome (Zan et al., 2025) using BWA (Li and Durbin, 2009). Single nucleotide polymorphisms (SNPs) were identified with the SAMtools mpileup pipeline (Li et al., 2009).

To ensure marker quality, SNPs were first filtered to retain sites with a mean sequencing depth greater than 2, a minor allele frequency (MAF) above 0.03, and missing rates below 0.5 for both individuals and loci (Poland et al., 2012; Lu et al., 2013). Missing genotypes were subsequently imputed using Beagle software (v5.1) (Browning and Browning, 2016). After filtering and imputation, a final set of 95,308 high-quality SNPs was retained for all subsequent analyses.

Genotype accuracy was evaluated by comparing GBS-derived genotypes (both before and after imputation) with whole-genome resequencing data from 28 individuals randomly selected from the study population. The mean SNP calling accuracy prior to imputation was 0.976, indicating high reliability of the raw GBS data. Following imputation, the average accuracy remained high at 0.960, suggesting that the Beagle imputation process did not introduce substantial bias.

### Genome-wide association study

Genome-wide association analysis was performed using the Genome-wide Complex Trait Analysis (GCTA, v1.94.1) software (Yang et al., 2011) with a mixed linear model association (MLMA) approach. A genomic relationship matrix (GRM) was constructed from the remaining high-quality SNPs to account for population structure and kinship. The MLMA model was specified as


y=Xb+Sɡ+Zu+e

where *y* is the vector of phenotypic observations, *b* is the vector of fixed effects, g represents the SNP effect being tested, *u* is the vector of random polygenic effects and *e* is the residual error. Genome-wide significance thresholds were determined using a Bonferroni correction based on the total number of SNPs (P = 1/N = 1.05 × 10^-5^).

### Regional heritability mapping

To quantify the contribution of different genomic regions to phenotypic variation at the whole-genome scale, regional heritability mapping (RHM) was employed to characterize the genetic architecture of the studied trait. Utilizing the high-quality genotype data, the genome was partitioned into 2-Mb sliding windows based on the average linkage disequilibrium (LD) decay, and a local genomic relationship matrix (local GRM) was constructed for each window.

To control for genome-wide polygenic background effects, a multi-component linear mixed model was fitted in which the local GRM corresponding to the focal window and a background GRM were jointly included. The background GRM was constructed using a leave-one-chromosome-out (LOCO) strategy; specifically, when analyzing windows on a given chromosome, all SNPs from the remaining chromosomes were used to build the background genomic relationship matrix.

### Construction of the genomic selection model

Genome-wide selection (GS) for tobacco tar content was conducted using 95,308 high-quality SNPs and the measured phenotypic values. Sixteen commonly used prediction models were applied, categorized as follows: (i) linear models, including ridge regression (RR), LASSO, and elastic net (EN), suitable for sparse genetic architectures; (ii) mixed and Bayesian models, including additive models (GBLUP, rrBLUP), non-additive models based on Reproducing Kernel Hilbert Space (RKHS, MKRKHS), and Bayesian approaches (Bayes A, Bayes B, Bayes C, Bayesian ridge regression, and Bayesian LASSO), accommodating a range of genetic effect distributions; and (iii) machine learning models, including deep neural networks (DNNGP), gradient boosting machine (GBM), random forest (RF), and support vector machine (SVM), capable of modeling complex non-additive and epistatic interactions. These 16 models, spanning linear, mixed/Bayesian, and machine learning approaches, represent widely used methods in contemporary genomic selection studies. Together, they account for diverse genetic architectures of tobacco tar content, providing a comprehensive framework for developing predictive models for this trait (Howard et al., 2014; Abdollahi-Arpanahi et al., 2020).

### Model evaluation and cross-validation

The predictive performance of the 16 GS models was assessed using five-fold cross-validation (k = 5) (Heslot et al., 2012). All models were implemented using the default hyperparameter settings provided by the corresponding R packages, following common practice in genomic selection benchmarking studies to ensure standardized comparison and to avoid dataset-specific overfitting. The population was randomly partitioned into five equal subsets; in each fold, 20% of the individuals (test set) were used for prediction, while the remaining 80% (training set) were used to train the models with both genotype and phenotype data. Five-fold cross-validation was performed once, and prediction accuracy was calculated across the five folds. Model performance was quantified using the Pearson correlation coefficient between predicted GEBV and observedphenotypes. Computational efficiency, including running time and resource usage, was also compared across models at varying sample sizes. The optimal GS model was selected based on a combination of prediction accuracy and computational performance. For computational benchmarking, larger sample sizes were generated by resampling with replacement from the original dataset, and these analyses were intended to illustrate relative computational trends.

### External validation of the genomic selection model

To evaluate the practical applicability and generalizability of the optimized GS model, an independent external validation was performed. An additional panel of 36 distinct tobacco accessions, designed to provide a balanced representation of population structure (comprising 12 flue-cured, 12 burley, and 12 sun-cured types), was cultivated alongside the main training population under the same environmental conditions. The phenotypic evaluation of tar content for these 36 accessions was conducted using the identical standardized smoking machine protocol as described above. Furthermore, genotypic data for this validation panel were generated using the same GBS pipeline and subjected to the identical SNP filtering criteria as the main training set. The GEBVs of these 36 accessions were predicted using the rrBLUP model trained on the initial 436 accessions. Model robustness was subsequently assessed by calculating the Pearson correlation coefficient between the predicted GEBVs and the observed field phenotypes.

## Results

### Genetic background of 436 tobacco germplasm accessions

In this study, GBS was performed on 436 tobacco accessions from the National Tobacco Germplasm Repository of China, covering diverse types including flue-cured, sun-cured and burley tobacco accessions (**Table 1**).

**Table 1:** Types and number of tobacco germplasm accessions used in this study.

Tobacco_Type	Origin	Class	Count
Burley	China	Cultivar	16
	China	Landrace	9
	Introduced	Cultivar	25
	Introduced	Landrace	15
Flue-cured	China	Cultivar	44
	China	Landrace	55
	Introduced	Cultivar	30
	Introduced	Landrace	33
Sun-cured	China	Cultivar	17
	China	Landrace	187
	Introduced	Landrace	5

**Figure 1:**
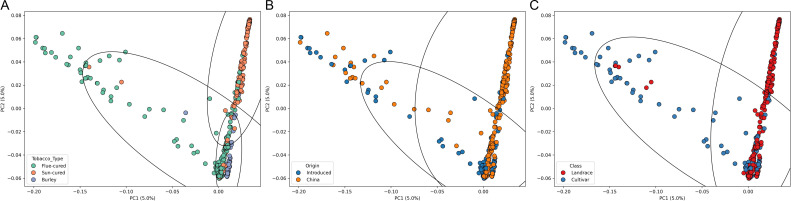
Genetic structure of 436 tobacco accessions. SNP-based principal component analysis (PCA). Different colors representing the genetic backgrounds according to tobacco type **(A)**, geographic origin **(B)**, and domestication stage **(C)**.

We analyzed the genetic background of 436 tobacco accessions using 95,308 single nucleotide polymorphism (SNP) markers. Principal component analysis (PCA) revealed weak-to-moderate population structure, with the first principal component explaining approximately 5.0% of the total genetic variance. No clearly separated subpopulations were observed, and substantial overlap existed among different tobacco types, origins, and classes (**Figure 1**). Our results suggest that the domestication process, from landrace to cultivated varieties, represents a major source of genetic differentiation, forming a continuous gradient rather than discrete subpopulations (**Figure 1**). Compared with other tobacco types, Burley exhibits a narrower genetic background (**Figure 1A**), consistent with its historical origin, as it arose from a single mutant of Maryland tobacco. In contrast, flue-cured tobacco shows a broader genetic background than Burley and sun-cured types. Geographic analysis further revealed that some Chinese and introduced U.S. accessions share highly similar genetic backgrounds (**Figure 1B**), reflecting the historical breeding process in China: before the 1950s, breeding primarily relied on introduced varieties, and many cultivated cultivars were derived from systematic selection of these introductions.

### Tobacco tar content distribution and heritability estimation

**Figure 2:**
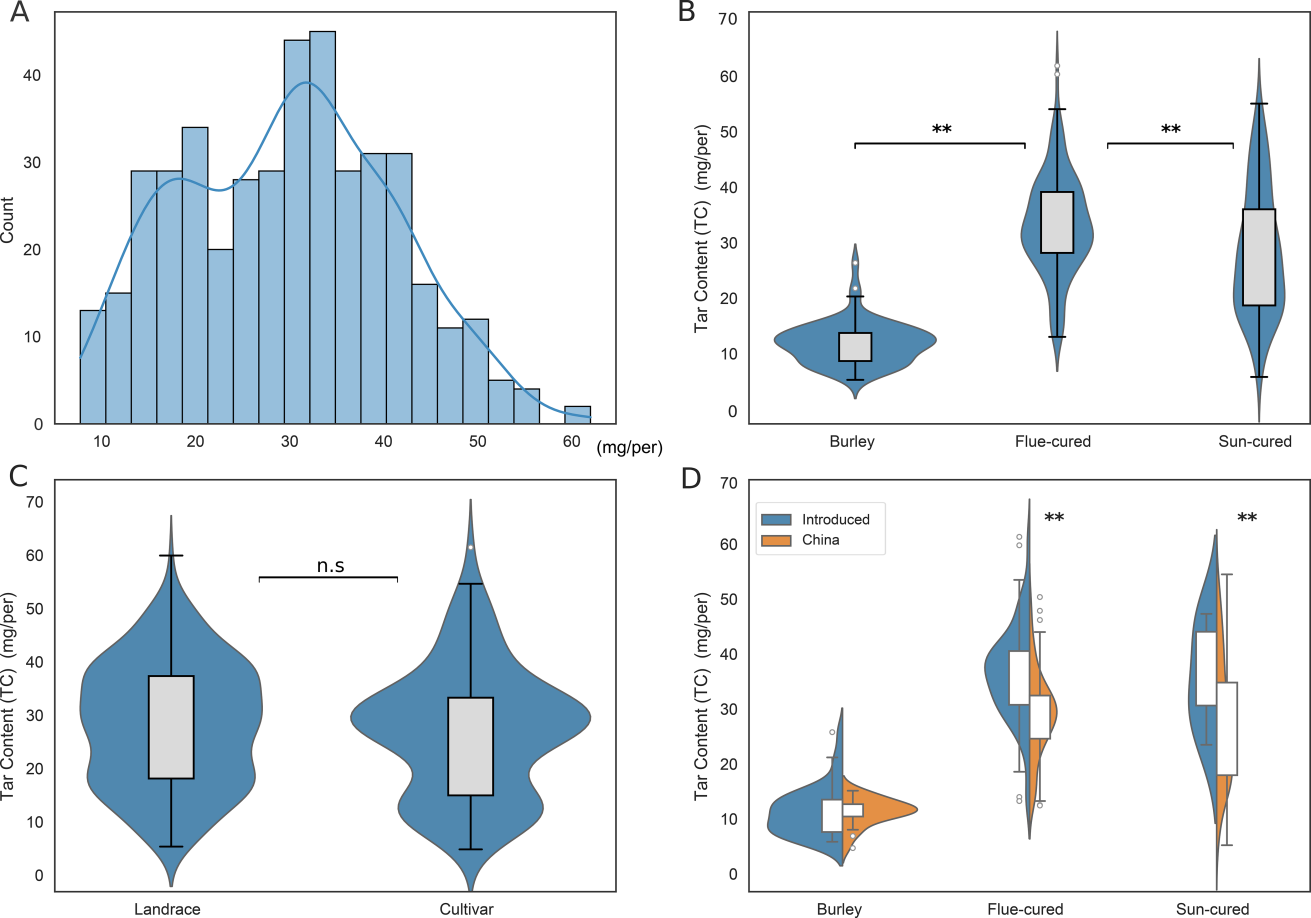
Phenotypic variation of tar content among 436 tobacco accessions. **(A)** Distribution of tobacco tar content. **(B)** Comparison of tar content among tobacco types. **(C)** Comparison of tar content between landrace and cultivated accessions. **(D)** Tar content variation by geographic origin. The error bars in the internal boxplots of the violin plots represent 1.5 times the interquartile range.

Phenotypic analysis of the tobacco tar content in 436 tobacco accessions indicated that the tobacco tar content approximately follows a normal distribution (**Figure 2A**), with a range from 7.6 to 61.8 mg/cigarette and an average of 29.7 mg/cigarette. About 70% of the samples were concentrated within the 20.0-40.0 mg/cigarette range. Among these, the tobacco tar content of the major tobacco cultivars in China, K326 and Yunyan 87, were 28.4 mg/cigarette and 33.9 mg/cigarette, respectively. We compared tar content among tobacco types and found that Burley tobacco averaged 14.0 mg/cigarette, significantly lower than flue-cured (34.3 mg/cigarette) and sun-cured (29.4 mg/cigarette) tobaccos (**Figure 2B**). No significant differences were observed between landrace and cultivated accessions (**Figure 2C**). In contrast, introduced germplasm, primarily from the United States, exhibited significantly higher tar content than Chinese accessions, except for Burley (**Figure 2D**). These findings reveal substantial phenotypic variation in tobacco tar content across both types and geographic origins.

The narrow-sense heritability of tobacco tar content was estimated at *h*^2^ = 0.70 ± 0.08. Likelihood ratio tests indicated a highly significant genetic effect (LRT = 197.9, df = 1, P < 10^-100^), suggesting that approximately 70% of the phenotypic variation is attributable to genetic factors. These results indicate that tobacco tar content is predominantly genetically determined, providing substantial potential for genetic improvement.

### Tar content appears to exhibit a predominantly polygenic genetic architecture

**Figure 3:**
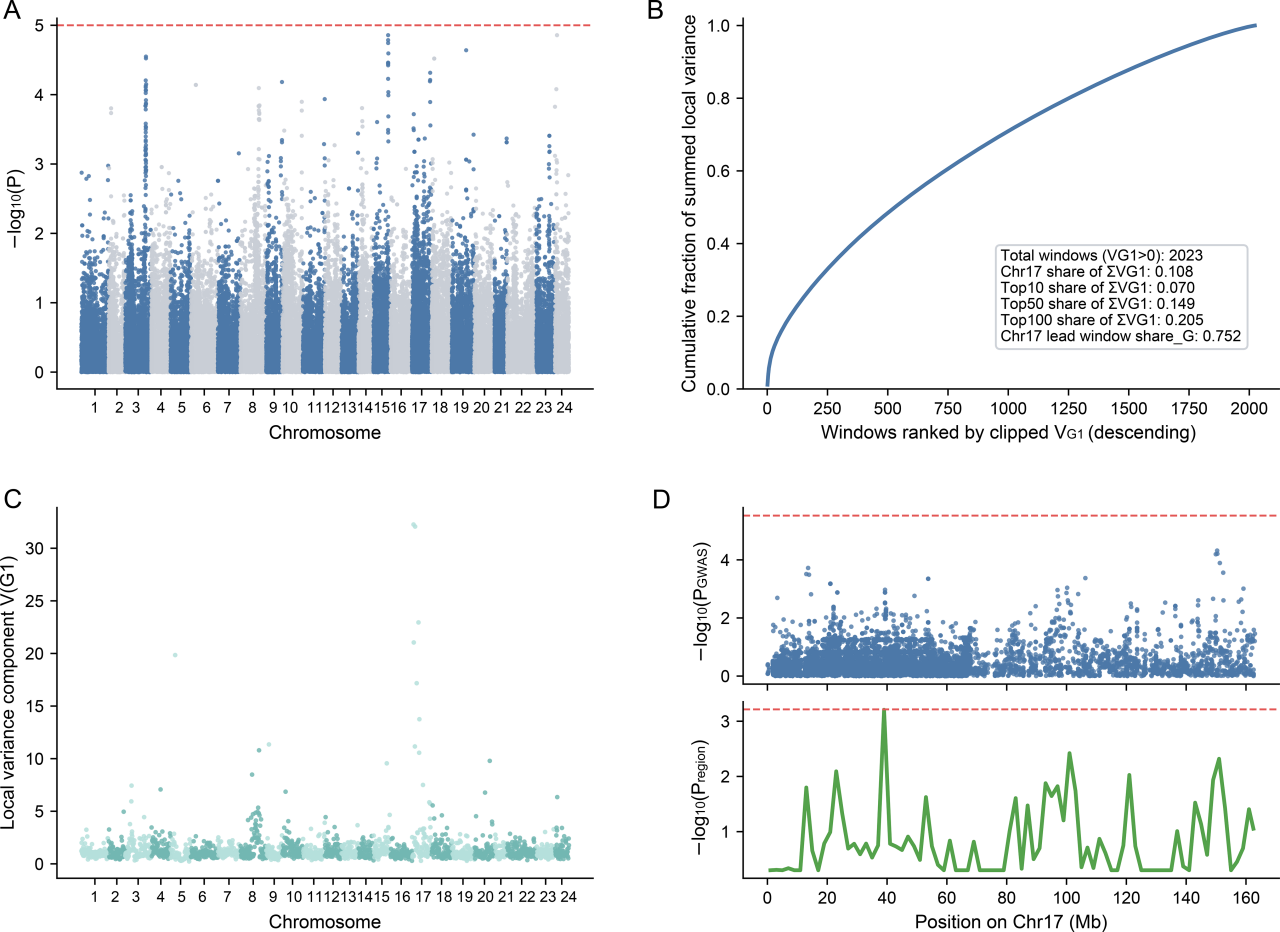
GWAS analysis of tobacco tar content. **(A)** Manhattan plot, the x-axis represents the genomic position across all chromosomes (color-coded by chromosome), and the y-axis shows the association significance [-log_10_(*p*-value)]. The red dashed line indicates the genome-wide significance threshold (*p* < 1.05×10^-5^, 1/N). **(B)** Genomic distribution of local variance components (*V_G1_*) estimated by RHM, highlighting a peak on Chromosome 17. **(C)** Cumulative fraction of total local genetic variance components (ΣVG1) ranked by genomic windows. The inset box details the variance proportions. **(D)** Fine-scale visualization of the significant region on Chromosome 17, aligning the GWAS -log_10_(*p*_GWAS_) values (top panel) with the RHM -log_10_(*p*_region_) values (bottom panel) across the physical positions. Dashed lines indicate their respective significance thresholds.

To further investigate the genetic basis of tar content in tobacco, we conducted a genome-wide association study (GWAS) using phenotypic data from 436 tobacco accessions. Genome-wide association analysis failed to identify any SNP surpassing the Bonferroni corrected significance threshold (*p* < 1/N = 1.05 × 10^-5^), indicating the absence of major-effect loci underlying the trait. Although modest enrichment of sub-threshold GWAS signals was observed on several chromosomes, including chromosomes 3,16 and 17, no individual variant exhibited a sufficiently strong effect to be detected at the single-marker level (**Figure 3A**).

Notably, the top ten SNPs ranked by association significance each explained only a small proportion of phenotypic variance, with *r*²ranging from 2.12% to 3.90% (**Supplementary Table 1**), further supporting the lack of dominant loci and suggesting dispersed small-effect genetic control.

To capture aggregated genetic effects beyond individual SNPs, regional heritability mapping (RHM) was performed using 2-Mb sliding windows with a step size of 500 kb across the genome (**Figure 3B**). This analysis revealed substantial heterogeneity in local genetic variance components, with multiple genomic regions showing elevated regional signals, among which chromosome 17 exhibited the strongest enrichment. When windows were ranked by their local variance components (VG1), the top 10, 50, and 100 windows collectively accounted for 6.99%, 14.86%, and 20.53% of the summed local variance components (ΣVG1), respectively (**Figure 3C**). Notably, chromosome 17 alone contributed 10.77% of ΣVG1, indicating pronounced regional enrichment of polygenic signals (**Supplementary Table 2**).

In addition, the lead 2-Mb window on chromosome 17 (chr17:12,000,001–14,000,000) showed the highest model-internal genetic share, defined as VG1/(VG1+VG2), reaching 0.752 (**Figure 3D**). Under the leave-one-chromosome-out framework, this value reflects the relative contribution of the focal region compared with the remainder of the genome within the window-specific multi-component model, suggesting strong localized aggregation of small-effect variants. Consistent with this, direct comparison of GWAS and RHM profiles along chromosome 17 revealed that, despite the absence of genome-wide significant SNPs, multiple consecutive windows displayed elevated likelihood ratio statistics that coincided with regions showing mild GWAS signal enrichment (**Figure 3D**).

Taken together, these results indicate that genetic effects on chromosome 17 arise from the collective contribution of numerous linked variants with small individual effects rather than from a single major-effect locus. More broadly, they support a predominantly polygenic genetic architecture characterized by dispersed genome-wide small-effect loci together with localized polygenic enrichment, with chromosome 17 representing the most prominent example of such aggregated genetic effects.

### Construction of a GS model for predicting tobacco tar content

**Figure 4:**
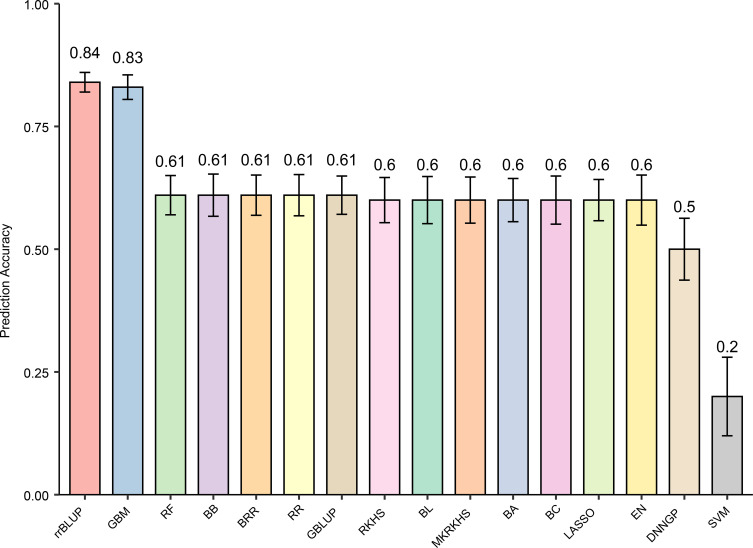
Prediction accuracy of tobacco tar content.

In this study, we constructed genome-wide selection (GS) models using genotypic and tar content phenotypic data from 436 tobacco accessions and evaluated the predictive performance of 16 GS models (Bayes A, Bayes B, Bayes C, BL, BRR, GBLUP, rrBLUP, Lasso, GBM, EN, RR, RKHS, SVM, MKRKHS, RF, and DNNGP). Except for SVM and DNNGP, all models achieved prediction accuracies ranging from 0.6 to 0.8. Among them, rrBLUP exhibited the highest accuracy at 0.84, followed closely by GBM with an accuracy of 0.83 (**Figure 4**, **Supplementary Table 3**). The relatively lower accuracy of SVM likely stems from challenges in detecting small-effect polygenic signals under high-dimensional settings with limited samples, while DNNGP may be constrained by overfitting given the moderate sample size. Under random cross-validation in this population, rrBLUP consistently showed the strongest predictive performance. However, we emphasize that model performance is dependent on population structure and validation strategy. In the present study, rrBLUP therefore represents a robust and effective approach for predicting tobacco tar content within this population.

### Comparison of computational speed and resource consumption of 16 genomic selection models

Computational efficiency and resource consumption are critical factors in large-scale breeding programs. To assess these factors, we conducted computer simulations comparing the runtime and resource usage of different GS models on large datasets. Simulations were performed on a laptop equipped with a 12th-generation Intel processor (12 cores, 2.50 GHz), 16 GB of RAM, running 64-bit Windows 11, and all analyses were executed within the R environment.

**Figure 5:**
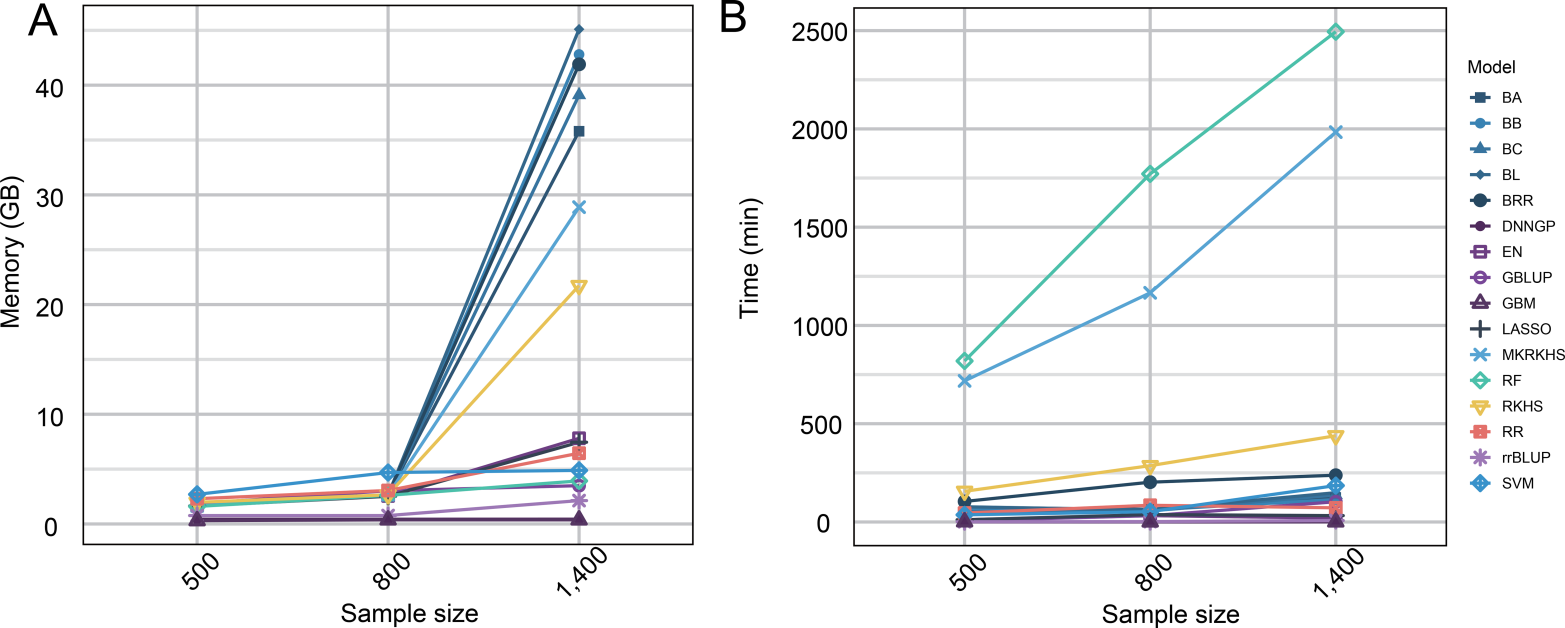
Comparison of operation speed and computing resources of different models. **(A)** The line plot compares the memory consumption (in gigabytes, y-axis) of the models. **(B)** The line plot shows the running time (in minutes, y-axis) required by different models (coded by color, see legend) across varying population sizes (x-axis).

Random sampling was used to simulate populations of 500, 800, and 1,400 individuals. To maintain realistic genetic structures, genotypic data were sampled with replacement from a dataset of 436 tobacco accessions. These resampling-based simulations were designed solely to illustrate relative computational trends among models under increasing sample sizes and do not represent true biological population expansion or genetic scalability. Each model was evaluated for runtime, memory usage, and CPU utilization using 5-fold cross-validation. Across all population sizes, rrBLUP exhibited superior computational speed (**Figure 5A**) and the lowest memory consumption (**Figure 5B**), requiring only 2.13 GB of memory for the largest population of 1,400 individuals.

### Validation of the prediction model using an independent field trial

**Figure 6:**
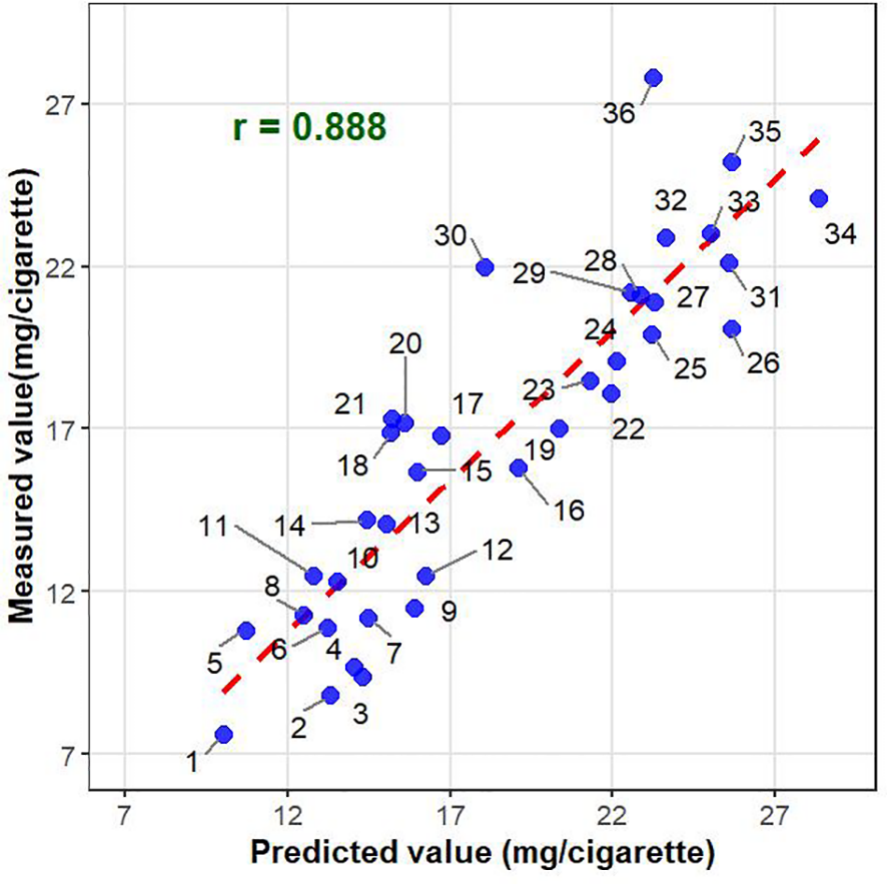
External validation of the rrBLUP genomic prediction model. The scatter plot illustrates the relationship between the genomic predicted values (x-axis) and the observed phenotypic tar content (y-axis) for 36 independent tobacco accessions.

To further validate the reliability and generalizability of the optimal prediction model, an external validation was conducted using an independent panel of 36 distinct tobacco accessions. To ensure a balanced representation of the population structure, this validation set comprised 12 flue-cured, 12 burley, and 12 sun-cured accessions. These materials were cultivated alongside the main training population, with their phenotypic data systematically evaluated under identical environmental conditions. Correlation analysis between the genomic predicted values and the observed phenotypic measurements yielded a highly significant Pearson correlation coefficient (*r* = 0.888). This strong concordance across different tobacco types demonstrates the strong robustness of the rrBLUP model, confirming its practical efficacy and structural stability for predicting tobacco tar content (**Figure 6**).

## Conclusion and discussion

In this study, we performed a comprehensive phenotypic and genomic analysis of tobacco tar content in 436 genetically diverse accessions, providing new insights into the genetic architecture of this economically and health-relevant trait. The phenotypic analysis revealed extensive variation in tar content across tobacco types and geographic origins, with narrow-sense heritability estimated at 0.70, indicating that a substantial proportion of the variation is genetically determined. These findings suggest significant potential for genetic improvement of tar content.

However, genome-wide association analysis did not identify any major-effect loci for tar content in this study, and even the top ten SNPs individually explained only 2.12-3.90% of the phenotypic variance, suggesting a predominantly polygenic architecture with dispersed small-to-moderate effects. Our regional heritability mapping analyses provide further insight into the genetic architecture of tobacco tar content. Rather than revealing discrete major-effect loci, RHM highlights localized enrichment of polygenic signals, most prominently on chromosome 17. Importantly, this enrichment reflects aggregation of numerous small-effect variants within specific genomic regions, rather than an additive partitioning of genome-wide genetic variance, as overlapping sliding windows and correlated genomic relationship matrices inherently preclude such decomposition. This interpretation is consistent with the absence of genome-wide significant SNPs in GWAS and the presence of spatially clustered but individually weak association signals. Together, these results indicate that variation in tar content is driven by dispersed genome-wide effects combined with regionally concentrated polygenic contributions, reinforcing a highly polygenic genetic architecture. This highly polygenic architecture not only renders traditional marker-assisted selection inefficient but also makes the discovery of candidate genes for tar content substantially more challenging compared with other tobacco traits such as nicotine or other agronomic traits (Liu et al., 2022; Yuan et al., 2023).

By evaluating 16 genomic selection models, we identified rrBLUP as the most accurate and computationally efficient model for predicting tar content. Notably, rrBLUP maintained high prediction accuracy (0.84) and low resource consumption even at large simulated population sizes, outperforming other linear, Bayesian, and machine learning models. The superior performance of the rrBLUP model is consistent with our GWAS and RHM findings. Because tobacco tar content is governed by a highly polygenic architecture characterized by numerous minor-effect loci rather than sparse major genes, rrBLUP—which mathematically assumes all markers contribute uniformly to the genetic variance—efficiently captures these genome-wide additive effects better than models assuming sparse architectures (e.g., LASSO) or those focused on complex epistatic interactions (e.g., SVM).

Furthermore, while internal cross-validation is valuable for model screening, it can sometimes inflate prediction accuracy due to relatedness leakage between training and testing sets in structured populations. To rigorously address this and confirm the practical breeding value of our model, we evaluated the optimized rrBLUP model on the completely independent external panel of 36 accessions. The model achieved an exceptionally high prediction accuracy (*r* = 0.888) across a balanced representation of flue-cured, burley, and sun-cured types evaluated under identical environmental conditions. This strong external validation strongly demonstrates that the rrBLUP model effectively captures the true additive genetic variance underlying tar content, rather than merely exploiting population structure, thereby proving its reliability for breeding applications.

Although genomic selection has achieved considerable success in other plant and animal breeding programs (Meuwissen et al., 2016; Wang et al., 2020), its application in tobacco remains very limited. Our study extends these findings by systematically comparing a broader panel of models, including deep learning approaches, and incorporating computational efficiency metrics, which are an important consideration for practical breeding applications.

The establishment of a robust GS framework for tar content provides a powerful tool to accelerate the breeding of low-tar tobacco varieties. By enabling accurate prediction of breeding values based solely on genotypic data, this approach reduces reliance on labor-intensive and costly phenotyping, which has historically limited the scale of low-tar selection. However, the practical application of GS in tobacco breeding still requires validation in independent populations and integration with multi-omics data to dissect regulatory networks underlying tar production. For instance, integrating transcriptomic and metabolomic profiles could help identify key genes and metabolites (e.g., those involved in phenylpropanoid or glycolytic pathways) that directly influence tar precursors.

In conclusion, our study demonstrates that tobacco tar content is a highly heritable trait and provides an optimized GS model with high prediction accuracy and computational efficiency. These findings offer both theoretical insights into the genetic architecture of tobacco tar and practical guidance for implementing genomic selection in low-tar breeding programs, complementing and expanding upon previous studies in the field.

## Data Availability

The datasets presented in this study can be found in online repositories. The names of the repository/repositories and accession number(s) can be found below: https://www.ncbi.nlm.nih.gov/, PRJNA936601.
